# Pan-Genome-Assisted Computational Design of a Multi-Epitopes-Based Vaccine Candidate against *Helicobacter cinaedi*

**DOI:** 10.3390/ijerph191811579

**Published:** 2022-09-14

**Authors:** Saba Ismail, Noorah Alsowayeh, Hyder Wajid Abbasi, Aqel Albutti, Muhammad Tahir ul Qamar, Sajjad Ahmad, Rabail Zehra Raza, Khulah Sadia, Sumra Wajid Abbasi

**Affiliations:** 1 Department of Biological Sciences, National University of Medical Sciences, Rawalpindi 46000, Pakistan; 2 Department of Biology, College of Education (Majmaah), Majmaah University, Al-Majmaah 11952, Saudi Arabia; 3 Pakistan Institute of Medical Sciences, Shaheed Zulfiqar Ali Bhutto Medical University, Islamabad 44000, Pakistan; 4 Department of Medical Biotechnology, College of Applied Medical Sciences, Qassim University, Buraydah 51452, Saudi Arabia; 5 Department of Bioinformatics and Biotechnology, Government College University Faisalabad, Faisalabad 38000, Pakistan; 6 Department of Health and Biological Sciences, Abasyn University, Peshawar 25000, Pakistan; 7 Department of Biosciences, COMSAT University, Islamabad 45550, Pakistan

**Keywords:** *Helicobacter cinaedi*, pan-genome, vaccine candidates, epitopes, multi-epitopes’ vaccine, docking, molecular dynamics simulation

## Abstract

*Helicobacter cinaedi* is a Gram-negative bacterium from the family *Helicobacteraceae* and genus *Helicobacter*. The pathogen is a causative agent of gastroenteritis, cellulitis, and bacteremia. The increasing antibiotic resistance pattern of the pathogen prompts the efforts to develop a vaccine to prevent dissemination of the bacteria and stop the spread of antibiotic resistance (AR) determinants. Herein, a pan-genome analysis of the pathogen strains was performed to shed light on its core genome and its exploration for potential vaccine targets. In total, four vaccine candidates (TonB dependent receptor, flagellar hook protein FlgE, Hcp family type VI secretion system effector, flagellar motor protein MotB) were identified as promising vaccine candidates and subsequently subjected to an epitopes’ mapping phase. These vaccine candidates are part of the pathogen core genome: they are essential, localized at the pathogen surface, and are antigenic. Immunoinformatics was further applied on the selected vaccine proteins to predict potential antigenic, non-allergic, non-toxic, virulent, and DRB*0101 epitopes. The selected epitopes were then fused using linkers to structure a multi-epitopes’ vaccine construct. Molecular docking simulations were conducted to determine a designed vaccine binding stability with TLR5 innate immune receptor. Further, binding free energy by MMGB/PBSA and WaterSwap was employed to examine atomic level interaction energies. The designed vaccine also stimulated strong humoral and cellular immune responses as well as interferon and cytokines’ production. In a nutshell, the designed vaccine is promising in terms of immune responses’ stimulation and could be an ideal candidate for experimental analysis due to favorable physicochemical properties.

## 1. Introduction

Antibiotic resistance (AR) is a phenomenon that appears when microorganisms such as bacteria, viruses, and fungi become resistant against a specific group of antibiotics [1,2]. It is mostly caused by the inappropriate use of antibiotics in human and animal medicine as well as in the environment and in agriculture. It has become a leading cause of mortality and morbidity worldwide, resulting in substantial economic losses. The principle behind AR is based on concept of evolution [3,4]. According to this theory, the adaptation of new strategies in therapeutics is necessary to alleviate the threat posed by AR [5,6]. One of these strategies involves the boosting of the immune system of humans through immunotherapeutic and immunological interventions. Bacterial infections quickly exhaust the natural defense mechanisms, limiting the therapeutic options available for acute therapy. Immunotherapeutic and immunological therapies can be used to combat such bacterial infections [7]. Furthermore, therapeutic monoclonal/polyclonal antibodies can be developed to generate vaccinations for certain pathogens to protect at-risk groups or to manage the diseases/infections due to AR [8]. There are currently no licensed immunoprophylactics or vaccines to combat nosocomial infections; however, implementing the aforementioned measures may assist in reducing illness load in hospitals [9].

*Helicobacter* spp. are divided into two groups: gastric (stomach) and enterohepatic (intestine and hepatobiliary). *H. pylori* is the most common human infection in the gastrointestinal group [10]. The gastrointestinal and hepatobiliary systems of diverse mammalian and avian hosts are mostly inhabited by enterohepatic species [11]. *Helicobacter cinaedi* is one of the best researched enterohepatic *Helicobacter* species that causes infections in humans. In 1984, *Helicobacter cinaedi* was discovered from rectal cultures of homosexual men for the first time. *H. cinaedi* has been isolated from both immunocompromised and immunocompetent people all over the world in the recent three decades [12]. It was once identified as a Campylobacter-like organism type-1 (CLO-1) until being reclassified as *H. cinaedi* in 1991. It is a spiral-shaped, Gram-negative enterohepatic bacillus found mostly in the digestive systems of humans and other animals [13,14]. According to several previously investigated reports, immunocompromised individuals are more susceptible to infection by *H. cinaedi*. Recent research has shown multiple examples of immunocompetent people infected with *H. cinaedi* [13,15].

In immunocompromised individuals, particularly those with rheumatoid arthritis and malignant lymphoma, several cases of *H. cinaedi*-related infections have been found [16,17]. *H. cinaedi*-related bacteremia was found in immune-competent people who had a hepatic cyst infection, carotid atherosclerosis, a thyroid infection presenting with thyroid storm, and a case of atypical Raynaud disease. A situation of *H. cinaedi* unique cellulitis was documented, as well as an *H. cinaedi*-caused vertebral osteomyelitis identified using 16S rRNA gene sequencing [18,19]. Anticancer chemotherapy and systemic steroids have been demonstrated to be independent risk aspects for persistent *H. cinaedi*-induced bacteremia; nevertheless, data suggest that targeted digestive cleansing with kanamycin might be an effective way to prevent infectious bacteremia from recurring [19]. This strain is hard to recognize at the species level. It was also discovered in tiny slush runs in wastewater treatment plants, indicating the dangers of activated sludge to human and environmental health. It also has a stronger vascular affinity than other *Helicobacter* species and appears to be associated to heart problems including arrhythmia and atherosclerosis [17]. *H. cinaedi* is known to cause many types of infections including diarrhea, gastroenteritis, fever, abdominal pain, arthritis, and neonatal meningitis in humans [18].

This study was designed to obtain insights about antigenic determinants of *H. cinaedi* and pinpoint all antigenic potential targets to design a multi-epitope, peptide-based vaccine. The failure to find a vaccination yet for the disease has further added to the severity of the AR problem. Furthermore, the absence of effective preventative measures and the lack of a treatment might lead to a rise in mortality and morbidity. To increase the maximal antibody formation and long-lasting immunological responses, immunoinformatics methods were used to combine epitopes to create a multi-epitope peptide, which was then adjuvanted to a suitable cholera toxin B subunit (CTBS) adjuvant [20]. The vaccine design was also put to a blind docking experiment to determine the design’s best possible binding mode to the rapid innate immune receptor TLR 5 (Toll-like receptor 5) receptor molecule. The complex was then employed in MD simulations to better comprehend complex structure dynamics and biological function. Finally, the complex’s binding free energies were calculated to confirm intermolecular affinity.

## 2. Methodology

**Figure 1:**
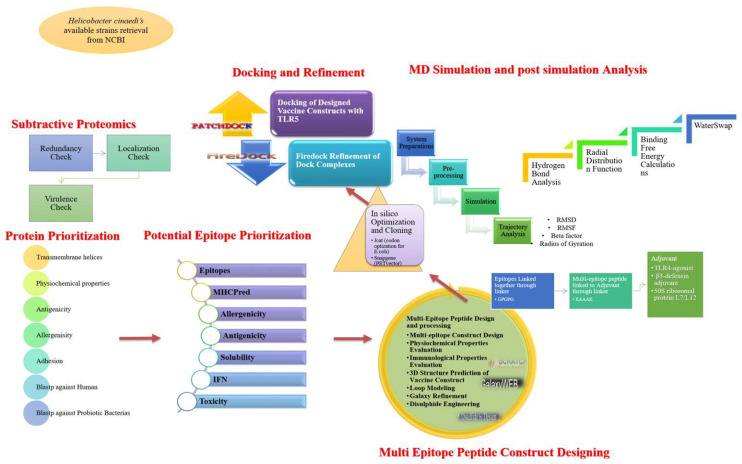
Schematic representation of computational approaches utilized in this study to design a vaccine construct against *H. cinaedi*.

The flowchart of the comprehensive computational analysis performed in this study is presented in Figure 1.

### 2.1. Gene Analysis and Pan-Genome Exploration

The term “pan-genome” refers to the whole gene sequence, which is made up of dispensable genome families and the core genome. The central genome is crucial in each species and is largely responsible for bacterial development; but the auxiliary genome contains crucial genes for resistance, stress mechanisms, and strain pathogenicity. The pan-genome was evaluated using the genomes of all nine strains of *H. cinaedi*, which are available in the GenBank libraries of the NCBI database using BPGA (bacterial pan-genome analysis) [21]. By conducting pre-processing stages via BPGA by the USEARCH program, sequence data are built up for creating a sequence identity with a cutoff score of 50%. The assembled output is used to fabricate the incidence of ambiguous genes and new gene families; then, it is utilized to calculate the pan-genome outline.

### 2.2. Pre-Screening Phase

The study embarked on the retrieval of the complete proteome of *H. cinaedi* from the genome database of the NCBI. The next important step was to generate the core sequence. The pan-genome analysis was performed using the BPGA (accessed on 2 January 2022) tool to generate the core sequence. The core sequence file retrieved by the BPGA tool was taken and was exposed to the later filters. The core sequence was then further clustered through the CD-HIT web server [22] (accessed on 3 January 2022), which removed the redundancy from the core sequence. The percentage identity threshold was set at 0.5%. CD-HIT is the rapid, efficient, and extensively used program that clusters and compares protein or nucleotide sequences and removes sequences that are showing an identity greater than the threshold value. The non-redundant protein dataset was BLASTp [23] searched against the core virulence factor database (VFDB) [24], which included selected proteins with a sequence identity less than 30% and a bit score more than 100. Proteins were then further evaluated according to their subcellular localizations. This is the key step for screening attractive vaccine proteins. Proteins that are present at the surface or are expelled to the outer environment of the pathogen are crucial to design the vaccine because they come in repeated contact with the host. The pathogen’s antigenic determinants are easily identified by the host immune system, resulting in targeted immune responses. The subcellular localization of proteins was predicted through PSORTb 3.0 [25] (accessed on 8 January 2022), which is the localization prediction tool. It was used to short-list the proteins that were confined in the inner membrane, outer membrane, and periplasmic and extracellular spaces. The results were then cross-checked with the CELLO2GO [26] localization predictor tools.

### 2.3. Prioritization of Vaccine Candidates

Short-listed proteins were then explored for a transmembrane helices check. Only proteins with 0 or 1 transmembrane helix were chosen and analyzed for further examination. The protein sequences were then used in ExPASY ProtParam [27] (accessed on 10 January 2022), which permits the computation of several physical and chemical parameters for a given protein sequence. The pivotal variable assessed in this depiction was the instability index, which was set at 40; the sequences showing the instability index greater than 40 were considered to be as unstable. The proteins showing stability were further processed for molecular weight evaluation. Ideally, expedient and effectual targeted vaccines are deemed to have molecular weight > 110 kDa. VaxiJen 2.0 (accessed on 11 January 2022) was used to determine the antigenicity of the proteins, with bacteria as the target organism and a threshold of >0.5. Antigenicity refers to the ability to attach selectively to adaptive immunity products such as antibodies and T-cell receptors. The adhesive properties of the antigenic proteins that resulted were investigated. Adhesive proteins are potential vaccination targets because they facilitate bacterial attachment and adherence to host tissues, which is critical for microbial pathogenicity [28]. The antigenic proteins’ adhesive properties were predicted using Vaxign [29] (accessed on 15 January 2022) with a minimal default value of 0.5. The adhesive proteins were aligned with the proteome of probiotic bacteria to pool homologs and prevent the chance of helpful bacteria being accidentally inhibited [30]. To avoid inhibition against the bacteria that is beneficial, a BLASTp (accessed on 16 January 2022) search was conducted against probiotic bacteria including three Lactobacillus species: *Lactobacillus casei* (taxid: 1582), *L. rhamnosus* (taxid: 47715), and *Lactobacillus johnsonii* (taxid: 33959) using an E-value cutoff of 0.005. Further to that, a homology check against the mouse proteome (taxid: 10088) was performed using the same parameters. The proteins that were screened were next evaluated in the epitope prediction step, which identified B-cell-generated T-cell epitopes for the proteins. The proteins’ linear B-cell epitopes were initially predicted using BepiPred Linear Epitope Prediction 2.0 [31,32] (accessed on 18 January 2022) with a threshold of 0.5. The B-cell epitopes were then used to map T-cell epitopes in IEDB T-cell epitopes’ prediction tools, which helps researchers find subsequences that bind to MHC class I and II alleles. The IEDB-recommended 2.22 technique was used for prediction, and the peptides were sorted by percentile score. High-affinity binders were defined as those with a low percentile score. Following that, MHCPred 2.0 [33] (accessed on 22 January 2022) analysis was used to determine the binding affinity potential of screened B-cell-generated T-cell epitopes, with only those having IC50 values for DRB*0101 (16) less than 100 nM being evaluated. VirulentPred [34] (accessed on 24 January 2022) was used to revalidate the virulence of antigenic epitopes. VaxiJen 2.0 [35] was used to confirm the antigenicity of the identified epitopes. Allergic sequences were deleted using AllerTOP 2.0 [36] (accessed on 28 January 2022), an in silico allergen prediction method. Non-soluble epitopes were discarded through Protein-Sol, and the IFN-γ-inducing potential of soluble epitopes was evaluated via the IFN epitope server [37] (accessed on 2 February 2022). The IFN-γ inducer epitopes were investigated using ToxinPred (accessed on 5 February 2022).

### 2.4. Multi-Epitopes’ Peptide Design

Low immunogenicity is the main issue related to peptide vaccines that can be overcome by joining immune-dominant epitopes to construct an MEPVC and appropriate adjuvanting. The MEPVC contains a number of overlapping immune-dominant epitopes that are defined as an opportune strategy to inhibit bacterial infections. The selected epitopes were joined by Gly-Pro-Gly-Pro-Gly linkers [38,39]. Further sequences of adjuvant CTB [40] were included to the construct to make a finishing vaccine candidate, and a complete investigation of the subsequent sections was performed with it. With the assistance of 3Dpro of the Scratch [41] (accessed on 6 February 2022) protein predictor, the tertiary configuration of the construct was modeled. Loops in the configuration were molded and a subsequent configuration modification was completed via GalaxyRefine of GalaxyWeb (accessed on 8 February 2022). Disulfide bonds were proposed in the structure to increase the strength and support in dynamics understanding of the construct. Disulfide by Design 2.0 (accessed on 15 February 2022) was used for the disulfide production of the deliberate vaccine construct. Inverse translation was used to adjust the vaccine component sequence for codon use. It was performed using the Java Codon Adaptation Tool service [42] to create a higher expression of the cloned sequence, which was then quantified using the percentage of the GC content and the CAI, or codon adaptation index, which has a value of 1 in the model. Lastly, the cloning of the optimized vaccine construct was performed through SnapGene into a pET-28a(+) expression vector.

### 2.5. Host Immune System Simulation

An agent-based model, the C-ImmSim server, was used to complete the immunogenicity classification and immune response profiling of the vaccine construct. It forecasts immunological epitopes using a position-specific scoring matrix and utilizes machine learning to evaluate immunological interactions. At the same time, the C-ImmSim server manages immunological simulation for three slots, which represent three distinct mammalian anatomical areas: bone marrow, thymus, and tertiary lymph nodes. The time step of injection was 1 and the number of the antigen injection was 1000. Random seeds were 12,345. Host HLA selectin included DRB1 0101, DRB1 0101, A0101, B0702, and B0702. Other parameters were set to default.

### 2.6. Designed Vaccine Docking

For the intended chimeric vaccine construct with a suitable immune receptor, the molecular docking was executed to interpret construct similarity for a certain immune molecule. This evaluation was vibrant because high-affinity interactions among the immune receptor and vaccine construct led to extremely substantial immune reactions. A blind docking approach was employed to anticipate the genuine binding of the vaccine construct with TLR5 having PDB ID: 3IJOU recovered from the protein data bank. TLR5 is a transmembrane protein that belongs to the pattern recognition receptor (PRR) family. Its activation causes the intracellular signaling of NF-kB to function as well as the production of cytokines, which leads to an innate immune system activation and, eventually, long-term adaptive immunity against *H. cinaedi* (https://pubmed.ncbi.nlm.nih.gov/15122529/) accessed on 25 February 2022. Molecular docking was accomplished with an online PatchDock server [43] that permits the docking of two interrelating molecules. The input clustering RMSD was set to 4.0 and complex type by default. Docked complexes were instantly upgraded with FireDock [44]. For the rescoring and improvement of protein–protein docking solutions, FireDock is a proficient platform. Complexes with the lowest global energy were graded top in addition to their exposure to a binding approach and intermolecular interactions by means of UCSF Chimera 1.13.1 [45], Visual Molecular Dynamics 1.9.3 [46], and Discovery Studio Visualizer 17.2.0.16349 [47] software.

### 2.7. Vaccine–TLR5 Dynamics Analysis

The nominated complex was investigated in a 100 ns manufacturing run using molecular dynamics simulation [48,49]. To assess the vaccine construct’s affinity for the TLR5 receptor, the MD simulation test took a long time to complete. Furthermore, it was critical to establish that epitopes may stay accessible to the host immune system for identification and processing in order to elicit a sufficient response. Assistant model building using Energy Refinement was used to complete these levels. The Antechamber software [50] was used to produce the complicated system libraries and settings for the TLR5 and vaccine built during the system preparation phase. The complex was immersed in a TIP3P solvation box (size 12) [51] using the Leap module [52]. The ff14SB force field [53,54] was used to describe the system’s intermolecular interactions. The system was neutralized by adding 25 Na^+^ counter ions. The system preparation for the production run was the focus of the second pre-processing phase. First, system energy was minimized in this phase in the subsequent direction: energy minimization of hydrogen atoms, energy minimization of water box, minimization of entire system atoms, and minimization of non-heavy atoms. The system was then gradually heated to 300 K. Langevin dynamics were utilized to keep the system’s temperature stable. The system’s hydrogen bonds were restricted using the SHAKE algorithm [55]. Moving on, the complex was equilibrated for 100 ps using a 2 fs time step. Pressure equilibrium was achieved using an NPT ensemble [56]. During the system equilibrium phase, the system was allowed to equilibrate on a 1-nanosecond time frame. On a time scale of 2 fs, simulated trajectories of 100 ns were created throughout the production phase. The Berendsen algorithm [57] with an NVT ensemble was chosen for production [58]. The CPPTRAJ module [59] was used to examine different structural characteristics for examining complex stability.

### 2.8. Estimation of TLR5–Vaccine Free Energies

The MMPBSA.py [60] module in Amber20 [61] was used to calculate the binding free energy of MMPBSA for an MEPVC and TLR5. The ante-MMPBSA.py unit of Amber generated a parameter file for the receptor, complex, and peptide molecule. For the binding energy estimates, 100 frames were chosen and assessed from many simulated trajectories. The estimation of the free energy variation between the unsolvated and solvated phases was the overall goal of this study. Gbind, solv was simplified for calculating the free energy of the anticipated complex by using these three equations:

ΔG bind, solv = ΔG bind, vaccum + ΔG solv, complex − ΔG solv, ligand − ΔG solv, complex(1)

ΔG solv = ΔG electrostatic (∈80 − 1) + ΔG hydrophobic(2)

ΔG vaccum = ΔE molecularmechanics − TΔG normalmodeanalysis.(3)

The disintegration of the total free energy of the complex in separate residue was attained for highlighting the crucial stabilizing residues.

## 3. Results and Discussion

### 3.1. Vaccine Targets’ Identification

**Figure 2:**
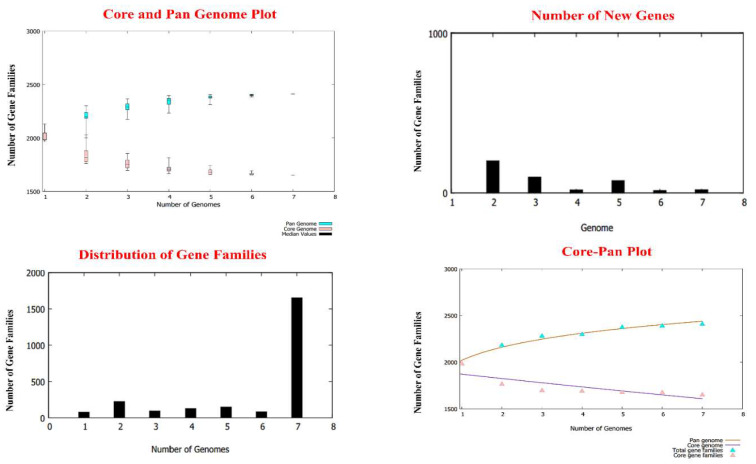
BPGA analyses of *H. cinaedi* genomes. The figure represents core and pan-genome plots of the bacterial genomes, as well as number of new genes in each strain of the pathogen, the distribution of gene families, and core-pan plot.

A total of 11,571 proteins have been identified in the core proteomes of nine strains of *H. cinaedi* bacteria that have been sequenced to date. The different pan-genome analyses of the bacteria is given in Figure 2. The core proteome was analyzed using a subtractive proteomics approach to predict potential vaccination candidates against selected bacteria. To eliminate redundant proteins, several CD-HIT analyses were performed. In bioinformatics, a redundancy check is unavoidable due to the large number of redundant databases that might induce biases and make the process computationally expensive [62]. This redundancy filter found 1675 non-redundant proteins in the core proteomic dataset that were represented separately. Non-redundant proteins’ subcellular localization predictions yielded 14 extracellular, 18 periplasmic, 9 outer membranes, 83 inner membranes, and 118 cytoplasmic proteins while other proteins were unknown and flagellar proteins. A total of 124 surface (periplasmic, extracellular, outer, and inner membrane) protein antigenic epitopes were actively identified by the host’s immune system, resulting in a targeted immune response. Furthermore, the pathogenicity of non-redundant proteins from the identified pathogen’s core proteome found in the exoproteome and secretome was investigated. Only 59 proteins out of 124 were discovered to be virulent, indicating that they are important regulators of bacterial pathogenesis and survival. Proteins with a molecular weight of 110 kDa have previously been found to be more effective for putative vaccine targets [63]. Out of all 59 proteins, 57 pathogenic proteins were discovered to have a molecular weight of less than 110 kDa and 39 of them had one or less than one TM helix, indicating that they should be studied further. Because of the difficulty in extracting, cloning, expressing, and purifying proteins containing numerous TM helices, they are rarely regarded as vaccine candidates [64]. The antigenicity of those 39 proteins was assessed further, out of which 16 proteins examined by the VaxiJen server were found to be antigenic. Allergenicity prediction indicated 13 of them were shown to be non-allergenic and 5 out of those 13 were adhesive, indicating that they may be used as vaccine targets. The NCBI database’s BLASTp program was used to identify and then remove human homologous proteins. Four human homologs were found and ruled out. Epitope mapping was then performed on these target proteins. The properties of selected proteins are listed in Table 1.

### 3.2. Epitopes’ Prediction

The 39 peptides were short-listed from the four vaccine candidates and tested for B-cell-derived T-cell prediction. Predicting B-cell epitopes is critical since the immune system’s protective mechanisms are activated when these epitopes bind to certain antibodies [65]. Afterward, these B-cell peptide sequences were examined for T-cell epitope prediction. CD8+ T-cells detect MHC I molecules on nucleated cell surfaces, causing presenting cells to die as a result of an immediate immunological response; on the other hand, MHC II molecules are found on antigen-presenting cells (APCs) and are recognized by CD4+ T-cells [66]. Among the four priority proteins, 79 B-cell-generated T-cell epitopes were chosen. Only epitopes with an antigenicity score of 0.4 were considered since they were thought to have the ability to bind antigen. After that, an additional MHCPred analysis was performed to find epitopes with the highest binding affinity to the DRB1*0101 allele [67], which is found in all Homo sapiens; epitopes that bind to this allele can induce significant immune responses. The IC50 value was used to calculate the binding capability. The lower the IC50 number is, the better the prediction quality is. We chose 105 epitopes with an IC50 of less than 100 nM.

**Figure 3:**
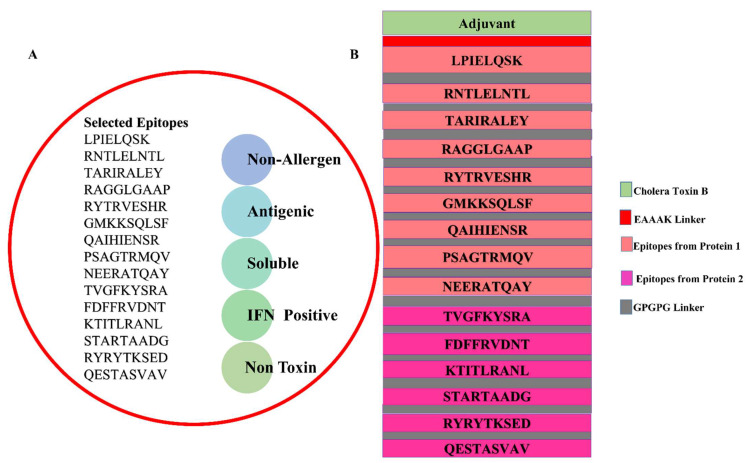
Selected epitopes that fulfill all the vaccine parameters and are used in multi-epitopes’ vaccine designing (**A**), and a schematic representation of epitopes’ ordering and adjuvant of the vaccine (**B**).

To rule out the possibility of allergic responses, allergenic peptide sequences were eliminated. This brought the total number of epitopes down to 62. Antigenic epitopes were evaluated for various physicochemical qualities to make epitope selection even more precise. The number of antigenic epitopes short-listed was 41. The epitopes were then subjected to a water solubility check, which allowed only the virulent peptides to be chosen; there were 37. The non-allergenic, non-toxic epitopes and water-soluble epitopes were further filtered for IFN-positive epitopes, which were 15. To eliminate vaccine-related potential toxicities, the epitopes were then tested for toxicity. The non-toxicity of all 15 nominated peptides was predicted. The final selected epitopes and schematic presentation of the designed vaccine construct is shown in Figure 3. Table 2 shows the final epitope selection from four priority proteins.

### 3.3. Physicochemical Properties of MEPVC

**Figure 4:**
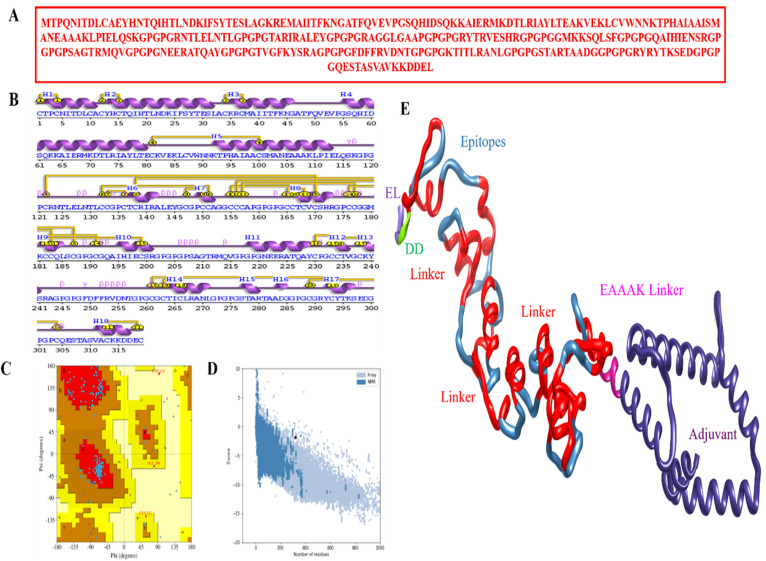
The sequence and structural analyses of a designed vaccine: (**A**) Vaccine’s primary amino acid sequence comprising linkers and adjuvant sequences. (**B**) Secondary structure elements of vaccine. The different secondary structure elements such as helix strand, beta turn, gamma turn, etc. can be seen. (**C**) Validation of constructed model of vaccine using Ramachandran plot (the Ramachandran plot colors can be interpreted at https://www.ncbi.nlm.nih.gov/pmc/articles/PMC5734310/) accessed on 28 February 2022. (**D**) Z-score. The black dot represents the vaccine model position on XY planes with respect to X-ray structures (light blue) and NMR structures (dark blue) of same size present in protein data bank (**E**) The vaccine’s 3D structure. Each element is highlighted in the figure.

To create an MEPVC, an AAY linker (used to avoid overlapping and maintaining stability) [39] was used to connect nine possible epitopes. The adjuvant beta-defensin was linked to the N-terminal of the proposed construct using the EAAAK linker [39]. The MEPVC had a final structure of 319 amino acids (Figure 4A). The physicochemical and immunogenic characteristics of the proposed MEPVC were next assessed. The MEPVC was highly antigenic (score of 0.93509) as well as non-allergenic. The design was also found to be thermally stable (28.48), and its small size had a molecular weight of 33.14, which will make it useful for testing. It had a GRAVY of −0.65 and a theoretical pI of 9.5, respectively. The MEPVC is hydrophilic if the GRAVY value is negative [68]. The MEPVC has a half-life of 30 h in mammalian reticulocytes, >20 h in yeast cells, and >10 h in *E. coli*, respectively. The MEPVC is very soluble, with a probability of 0.93509. The secondary structure of the MEPVC is shown in Figure 4B.

### 3.4. Vaccine Structure Prediction

The next stage was to use the Scratch Prediction server’s 3Dpro to predict a stable, 3D-structured MEPVC, which was then loop modeled using GalaxyLoop. The designed, complex had 11 loop modeling runs: Leu32-Pro54, Ala81-Asn9, Ser116-Pro135, Gly136-Pro147, Gly153-His172, Arg173-Gly192, Gln193-Arg212, Met227-GLY246, Pro247-Thr266, Leu267-Ala282, and Gly286-Gln306. The GalaxyRefine server was then used to fine-tune the loop-modeled construct. It was searched for both locally and globally, but with more constraints. In comparison to the input structure, Model 5 was chosen because it had a lower MolProbity (1.911), the lowest stable galaxy energy, a clash score (9.1), lower bad rotamers (0.8), and a higher number of Rama preferred residues (93.7). Ramachandran plot analysis was used to verify the final refined model’s validity [69]. The most favored region, additionally allowed region, generously allowed region, and disallowed region included 92.6%, 6.9%, 0.4%, and 0.0% of the total amino acids, respectively (Figure 4C). The Z-score of the vaccine was −1.8 (Figure 4D). The 3D structure of the MEPVC is given Figure 4E.

#### Disulfide Engineering

**Figure 5:**
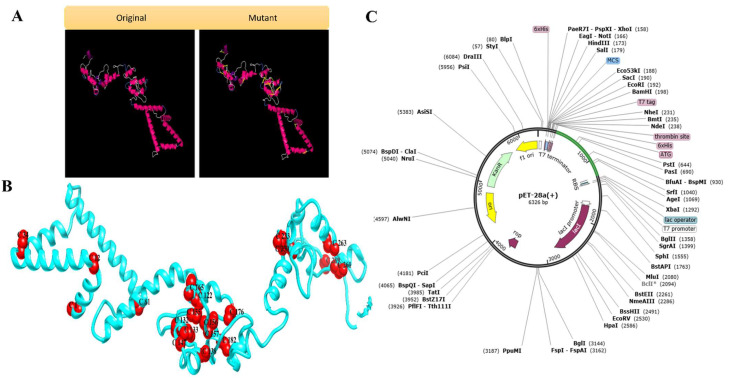
(**A**) Wild and mutant structures of designed vaccine. The yellow sticks are introduced disulfide bonds. (**B**) The disulfide residues are shown in the designed vaccine structure. (**C**) In silico cloned vaccine sequence in expression vector.

The redesigned vaccine design was then disulfide engineered to reduce conformational entropy, resulting in improved folded orientation stability [70]. Disulfide bonds were tested both within and between chains. Mutational potential was discovered in 28 pairs of residues. Because of their permissible energy values and Chi3 angles, 20 of these 28 pairs were chosen to be modified: Met1-Gln4, Glu12-Asn15, Gly34-Glu37, Ala81-Ile100, Gly122-Glu170, Gly132-Arg151, Pro133-Gly136, Ala138-Phe187, Pro147-Gly150, Leu155-Tyr166, Gly156-Ser183, Ala157-Pro177, Arg165-Arg168, Gly176-Pro191, Lys182-Asn199, Gly230-Gly234, Pro233-Phe238, Pro261-Arg293, Lys263-Thr266, Pro289-Gly304, Val313-Leu319s. Figure 5A shows the vaccine design’s original and mutant structures as well as the addition of disulfide links.

### 3.5. In Silico Cloning

The MEPVC vaccine was cloned and expressed within the expression vector using the Java Codon Adaptation Tool (JCat) [55]. In silico cloning was performed using the cDNA sequence obtained by reverse translation. JCat found a 0.99 CAI score and 56.88% GC content, indicating that the vaccine protein was highly expressed in the E. coli system. To clone the MEPVC gene into pET28a (+) plasmid for expression in E. coli, restriction sites were added to the 5 and 3 ends of the sequence and NdeI and XhoI enzymes were used. The sequence was cloned into the plasmid pET28a (+) using the SnapGene program. The clone had a size of 5849 bp. The disulfide-engineered MEPVC 3D structure and the cloned vaccine in the expression vector are shown in Figure 5B and Figure 5C, respectively.

### 3.6. Simulating Host Immune System

**Figure 6:**
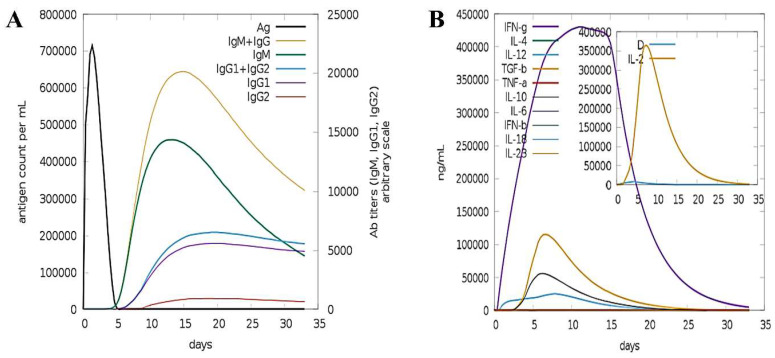
Immune simulation analysis of designed vaccine. (**A**) Different immunoglobulin count per mL simulated against antigen. (**B**) Level of interferon and interleukins in ng/mL to the antigen.

The MEPVC was examined for its immunogenicity and capability to induce immunity in the human body [71]. With a high level of the MEPVC antigen presentation to the host immune system over 5 days, a substantial rise in the secondary immune response generation in IgM + IgG type was seen. IgM levels, which indicate the major immunological response, were also found to be elevated. High levels of IgM, IgM + IgG, IgG1, IgG2, and IgG1 + IgG2 and a large B-cell population can be seen against vaccine in Figure 6A. Similarly, there was an exceedingly significant increase in IFN-g (>400,000 ng/mL) for nearly 33 days (Figure 6B).

### 3.7. Vaccine Docking with TLR5

**Figure 7:**
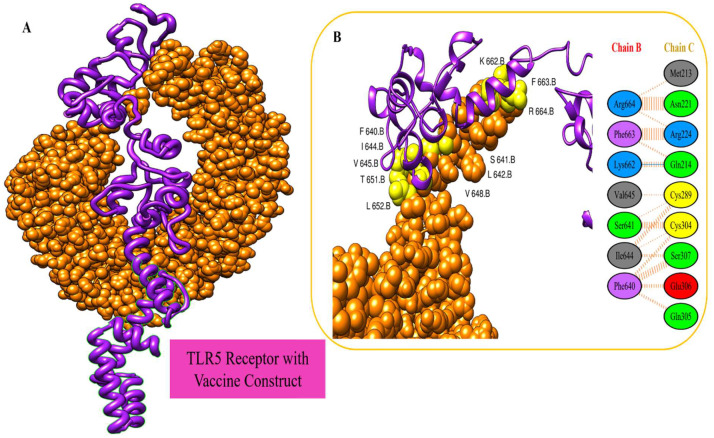
(**A**) Molecular docking of designed vaccine (in purple cartoon) with immune receptor (in orange cartoon). (**B**) Binding residues of vaccine–TLR5.

Using a molecular docking approach, the best docked vaccine pose to TLR5 immune receptor molecules was determined. The top 10 models of PatchDock [43] were selected based on the global energy score. For refining docked solutions, the FireDock server [44] was employed. The refined candidates were ordered according to their binding energies. The final model was picked from among the top 10 FireDock models based on the binding score. The MEPVC demonstrated robust interactions with human immunological receptors, according to the docking studies (Figure 7). Table 3 and Table 4 show PatchDock and FireDock docking solutions and associated docking scores, respectively.

### 3.8. Molecular Dynamic Simulation

**Figure 8:**
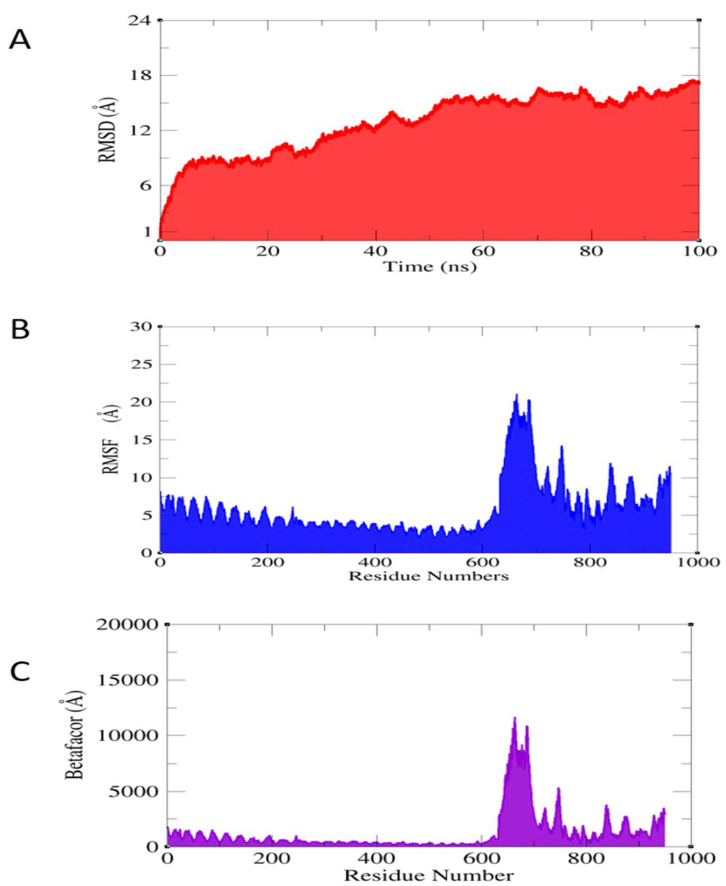
Exploring the dynamic behavior of vaccine–immune complex. (**A**) RMSD deviations, (**B**) RMSF residue fluctuation, and (**C**) Beta-factor thermal residue fluctuation.

The information obtained by docking provides valuable insight into the intermolecular docked conformation. Its ability to provide insights into its structural dynamics is limited. To better characterize the docked complexes, a molecular dynamics simulation was used. The C alpha atoms were explored for the divergence among the protein structure over the course of a 100 ns simulation. The complex’s stability was measured with root mean squared deviation (RMSD). As shown in Figure 8A, various structural changes were measured over the duration of the simulation. The highest RMSD noticed for the system was 15 Å, and the RMSD trend was seen in a steady increase. The vaccine upon trajectories’ analysis noticed the docked vaccine structure with TLR5 was very stable but the steady RMSD increase was due to a large percentage of system loops. The vaccine atomic residues’ flexibility and rigidity to the TLR5 target must be investigated since it gives a measure of atomic fluctuations. Throughout the simulation, this was estimated for the suggested complex structure with a mean square value of 3–5 Å. The highest RMSF was observed in the loop areas and at the lowest N and C terminals of the vaccine (Figure 8B). The β-factor was again calculated to revalidate RMSF, which gave the same residue range to show fluctuations (Figure 8C). Throughout the simulation, the vaccine molecule remained stable at the docking location. At each nanosecond, the vaccine docked to TLR4 was investigated, and no significant changes in the protein structure were detected.

### 3.9. Hydrogen Bond Analysis

**Figure 9:**
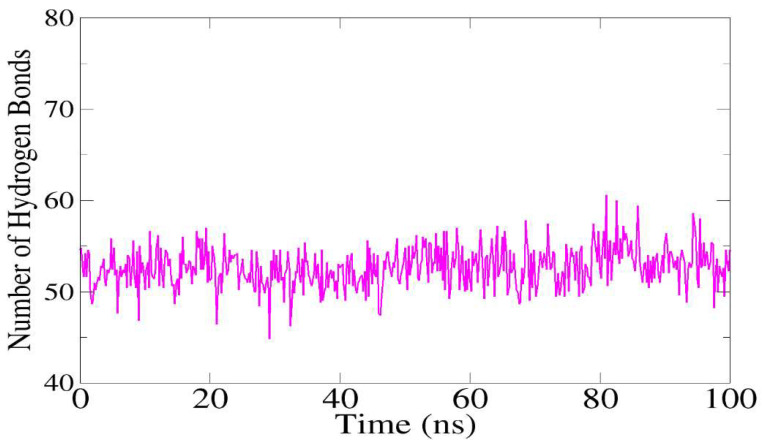
Number of hydrogen bonds verses simulation time.

The system became stable as a result of the key interacting residues dominating throughout the simulation run. A high number of hydrogen bonds was found to be formed between the vaccine and TLR5 (Figure 9). On average, in each frame, approximately 50 hydrogen bonds were formed among the residues of the vaccine and TLR5 mentioned in the docking section.

### 3.10. Binding Energy Calculations

The intermolecular binding TLR5 with the designed vaccine is important in the prospect of stimulating the host immune system and generating protective immune responses. As a result, in the current investigation, a simulated system was used to calculate binding free energies using the MMPBSA/MMGBSA methods. We calculated the complex’s binding free energies to determine the critical function of a chemical interaction at the atomic level. The MMPBSA and MMGBSA suggest that the majority of residues in the pocket areas had lower binding energies, with average values of −5.8 kcal/mol and −1.4 kcal/mol. As most of the binding energies of the residues move closer to the specific straight line, a linear regression value estimate is dependent on the time scale of the simulation intervals. Table 5 summarizes the entire set of energies for both methods considering the complex. Robust interactions between the receptor and vaccine were investigated in these studies. The following interactions were discovered as dominant for total system energy, including the Columbic interactions: (ΔEele = −156.97 kcal/mol), van der Waals energies (ΔEvdw = −391 kcal/mol), and non-polar solvation energy (ΔGnpol = −20.87 kcal/mol). The polar solvation energy of MMGBSA (ΔGsolv/GB) was 88.99 kcal/mol, while that of MMPBSA (ΔGsolv/PB) was 87.63 kcal/mol. MMGBSA (tot/GB = −479.88 kcal/mol) and MMPBSA (Δtot/PB = −484.15 kcal/mol) were the total binding energy calculations.

### 3.11. WaterSwap Energies’ Calculation

**Figure 10:**
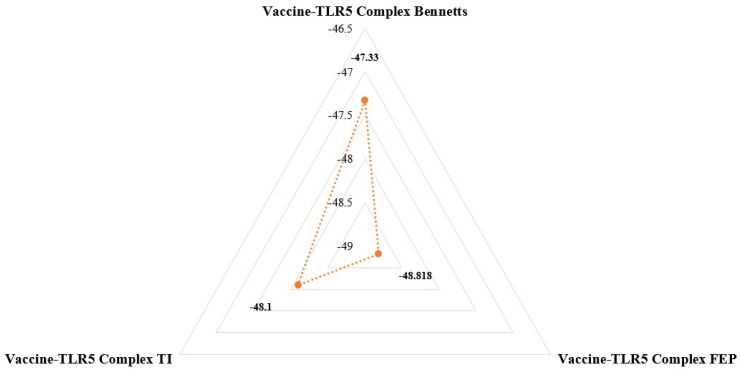
Binding free energy calculation using WaterSwap method. The energy values given are calculated in kcal/mol.

An innovative computational method called WaterSwap is used to calculate the absolute TLR5–vaccine binding free energies. WaterSwap considers the binding free energies of protein–ligand, ligand–water, and protein–ligand–water interactions, thus eliminating double decoupling problems of cavitation in solvent approaches. The total absolute binding free energy in the current situation was −48.08 kcal/mol, as calculated from three scoring functions: Bennett’s (−47.33 kcal/mol), FEP (−48.818 kcal/mol), and TI (−48.1 kcal/mol), as reported in Figure 10. As expected by MMPBSA and MMGBSA, the complex binding energy was well converged and relatively stable.

## 4. Conclusions

In this study, pan-genome and subtractive proteomic approaches were applied to short-list potential vaccine candidates against *H. cinaedi*. Four vaccine candidates (TonB dependent receptor, flagellar hook protein FlgE, Hcp family type VI secretion system effector, flagellar motor protein MotB) were identified as promising vaccine candidates capable of inducing immunity; they were subsequently subjected to an epitopes’ mapping phase. The predicted epitopes were passed through various immunoinformatics’ filters including antigenicity, allergenicity, toxicity, etc.; only promising epitopes were used in the MEPVC. The immunoinformatics tool, to prioritize the potential candidates for a vaccine, is an efficient method to determine cost-effective vaccine designing. The vaccine was found to efficiently dock with TLR5 and showed stable dynamics during the simulation time. The MEPVC was subjected to a few more analyses, confirming its efficacy and ability to induce immune responses against the *H. cinaedi*. Despite the fact that the study’s findings are encouraging, there are some limitations to the study. Experimental testing (wet laboratory) is required to determine the optimal order of epitopes in a vaccine creation so that the best potential combination can be achieved in the end. Furthermore, the findings of the current research call for the experimental validation of candidate vaccine constructs followed by in vitro and in vivo testing, in order to report vaccines that are safe, effective, and immunogenic against the malarial infection caused by *H. cinaedi* in the future.

## Figures and Tables

**Table 1:** Physicochemical properties of prioritized vaccine proteins.

Category of Proteins	Transmembrane Helices (TMHMM)	Physicochemical Properties						Antigenicity	Allergenicity	Adhesion	Human Blast
		Amino Acid	Molecular Weight	Gravy	Aliphatic Index	Instability Index	Theoretical PI				
**Outer membrane**											
>core/100/1/Org1_Gene1402 (TonB dependent receptor)	1	726	81.20	−0.42	76.17	40.6	8.75	0.60	Non	0.68	Non-significant
**Extracellular**											
>core/105/1/Org1_Gene1184 (Flagellar hook protein, FlgE)	0	718	77.15	−0.31	76.89	25.11	5.04	0.63	Non	0.82	Non-significant
>core/1610/1/Org1_Gene663 (Hcp family type VI secretion system effector)	0	171	18.85	−0.46	74.74	41.28	5.46	0.79	Non	0.59	Non-significant
**Inner membrane**											
>core/1187/1/Org1_Gene21 (Flagellar motor protein MotB)	1	250	27.68	−0.28	86.28	44.78	4.75	0.63	Non	0.50	Non-significant

**Table 2:** Selected epitopes filtered through different immunoinformatics analysis.

Protein	B-CELL	MHC II	P. Rank	MHC I	P. Rank	MHC-Pred	Score	Allergenicity	Antigenicity	Solubility	IFN	Toxinpred
>core/100/1/Org1_Gene1402	IMSELPIELQSKQI SVVEKKDLLQK	IMSELPIELQSKQIS	12.1	LPIELQSKQI	0.07	LPIELQSKQ	34.2	Non	Antigen	Soluble	Positive	Non-Toxin
	GRNTLELNTLDPY	GRNTLELNTLDPY	2.8	RNTLELNTL	7	RNTLELNTL	10.5	Non	Antigen	Soluble	Positive	Non-Toxin
	SSYNTQSDTFAATARIRALEYGS VNNLFGGRAEVLGGGGG	SDTFAATARIRALEY	9.1	ATARIRALEY		TARIRALEY	5.14	Non	Antigen	Soluble	Positive	Non-Toxin
	YTKDSTRYYTQGR YTRVESHRAGGLG AAPGSSYGILMSE DPISEY	RAGGLGAAPGSSYGI	6.4	RAGGLGAAP	11	RAGGLGAAP	98	Non	Antigen	Soluble	Positive	Non-Toxin
		QGRYTRVESHRAGGL	7.9	RYTRVESHR	0.19	RYTRVESHR	9.2	Non	Antigen	Soluble	Positive	Non-Toxin
	GMKKSQLSFAESME	GMKKSQLSFAESME	9.8	GMKKSQLSF	0.01	GMKKSQLSF	76	Non	Antigen	Soluble	Positive	Non-Toxin
	TISKTKSSTEQSNNNQAI HIENSRLLDENSVIHSGA	QSNNNQAIHIENSRL	1.2	QAIHIENSR	0.04	QAIHIENSR	12.7	Non	Antigen	Soluble	Positive	Non-Toxin
	NFKNPSAGTRMQVTP SGSSTLTIANPLIKP	KNPSAGTRMQVTPSG	3.7	NPSAGTRMQV	0.36	PSAGTRMQV	86.1	Non	Antigen	Soluble	Positive	Non-Toxin
	RGYGAKTRIDPNEE RATQAYTMT	IDPNEERATQAYTMT	20	NEERATQAY	0.06	NEERATQAY	23.1	Non	Antigen	Soluble	Positive	Non-Toxin
>core/105/1/Org1_Gene1184	TVGFKYSRASFV	TVGFKYSRASFV	1.3	TVGFKYSRA	3.3	TVGFKYSRA	77.6	Non	Antigen	Soluble	Positive	Non-Toxin
	QGWVRPPLEAAESGTMSD FDFFRVDNTGPVRNIQIDP GMVMPARATKTITLRANL NAGRHIDQMQEIAALDST ARTAADGVAPVYDSRGVLMQ	FDFFRVDNTGPVRNI	7.6	FDFFRVDNT	15	FDFFRVDNT	24	Non	Antigen	Soluble	Positive	Non-Toxin
		KTITLRANLNAGRHI	0.79	KTITLRANL	0.5	KTITLRANL	6.7	Non	Antigen	Soluble	Positive	Non-Toxin
		AALDSTARTAADGVA	6	STARTAADGV	0.69	STARTAADG	63.2	Non	Antigen	Soluble	Positive	Non-Toxin
	AFRYRYTKSEDADSTTGQF RTTEDLRALIQYDANM IKNPEKNYQESTASVAV	AFRYRYTKSEDADST	6.9	RYRYTKSEDA	1.7	RYRYTKSED	12.4	Non	Antigen	Soluble	Positive	Non-Toxin
		NPEKNYQESTASVAV	9.6	QESTASVAV	0.13	QESTASVAV	21.8	Non	Antigen	Soluble	Positive	Non-Toxin

**Table 3:** PatchDock docked solutions-based docking score.

Solution No.	Score	Area	Atomic Contact Energy	Transformation
1	23,492	3959.3	113.97	3.06 0.83 −2.27 81.63 43.88 59.96
2	20,000	3297.6	123.73	0.83 0.47 2.98 111.75 118.06 137.80
3	19,574	3202.6	−374.66	−2.85 0.25 −0.50 167.15 133.05 74.02
4	19,538	2663.1	198.87	−1.60 0.59 −2.94 108.59 1.32 137.65
5	19,298	4046.2	−470.53	1.04 −1.25 −0.94 54.91 39.29 111.78
6	18,774	3557.9	100.44	−1.03 0.12 −0.90 123.16 105.31 143.35
7	18,682	2483.8	429.62	2.85 −0.58 −2.65 118.04 91.65 57.75
8	18,588	2610.5	242.81	0.26 −0.30 −1.31 71.64 60.30 139.08
9	18,408	3281.2	−107.39	2.61 0.60 −1.66 88.02 60.20 61.85
10	18,264	3714.8	353.54	−1.37 0.59 2.51 65.97 19.97 137.43
11	18,174	3498.7	−227.03	2.12 0.23 −0.99 75.52 54.46 52.60
12	18,080	2482.3	234.36	0.12 −0.59 −1.43 76.73 53.89 133.59
13	17,852	2598.1	430.16	−1.31 0.64 1.86 62.13 59.69 124.78
14	17,564	2876.2	166.54	−2.56 0.22 −2.35 97.84 68.44 93.37
15	17,558	3028.1	95.72	−3.13 0.94 −2.51 75.37 44.11 61.76
16	17,494	2829.5	243.03	2.91 0.39 0.29 111.91 80.87 72.90
17	17,466	2496	376.63	−0.02 0.60 0.18 81.40 43.87 182.40
18	17,434	2112.7	412.27	1.90 −0.36 0.69 114.91 56.10 60.34
19	17,408	2299.3	456.21	−0.88 −0.19 2.51 88.98 92.68 116.39
20	17,276	2243.5	162.58	2.76 −0.86 −0.46 49.05 99.23 33.79

**Table 4:** Top 10 docked solutions of FireDock.

Rank	Solution Number	Global Energy	Attractive van der Waals Energy	Repulsive van der Waals Energy	Atomic Contact Energy	Hydrogen Bonding Energy
1	3	−13.7	−13.19	5.03	−7.61	−0.83
2	10	8.40	−14.75	13.32	8.68	−1.62
3	2	12.38	−9.09	3.37	7.04	−2.23
4	9	13.54	−11.29	18.74	6.08	−1.61
5	8	30.08	−19.11	58.67	9.00	−2.15
6	4	64.05	−18.81	101.92	14.73	−3.36
7	1	165.6	−20.49	203.14	8.92	−1.40
8	5	200.3	−26.97	236.17	3.87	−1.96
9	7	652.5	−33.22	873.36	15.34	−7.18
10	6	1300.1	−39.65	1620.54	20.31	−7.70

**Table 5:** Intermolecular binding free energies in kcal/mol.

Energy Parameter	TLR5–Vaccine Complex
**MMGBSA**	
Van der Waals (ΔEvdw)	−391.03
Electrostatic (ΔEele)	−156.97
Polar (ΔGsolv/GB)	88.99
Non-polar (ΔGnpol)	−20.87
Gas phase	−548
Solvation	68.12
Net (tot/GB)	−479.88
**MMPBSA**	
Van der Waals (ΔEvdw)	−391.03
Electrostatic (ΔEele)	−156.97
Polar (ΔGsolv/PB)	87.63
Non-polar (ΔGnpol)	−23.78
Gas phase	−548
Solvation	63.85
Net (Δtot/PB)	−484.15

## Data Availability

The data presented in this study are available within the article.
